# Supplementary material for: Data Representation Bias and Conditional Distribution Shift Drive Predictive Performance Disparities in Multi-Population Machine Learning
Source: bioRxiv. 2026 May 28:2025.06.18.658431. Preprint. [Version 2] doi: 10.1101/2025.06.18.658431 (PMC13232287; doi:10.1101/2025.06.18.658431)
Supplement: Supplement 1 [file NIHPP2025.06.18.658431v2-supplement-1.pdf]

Supplemental information

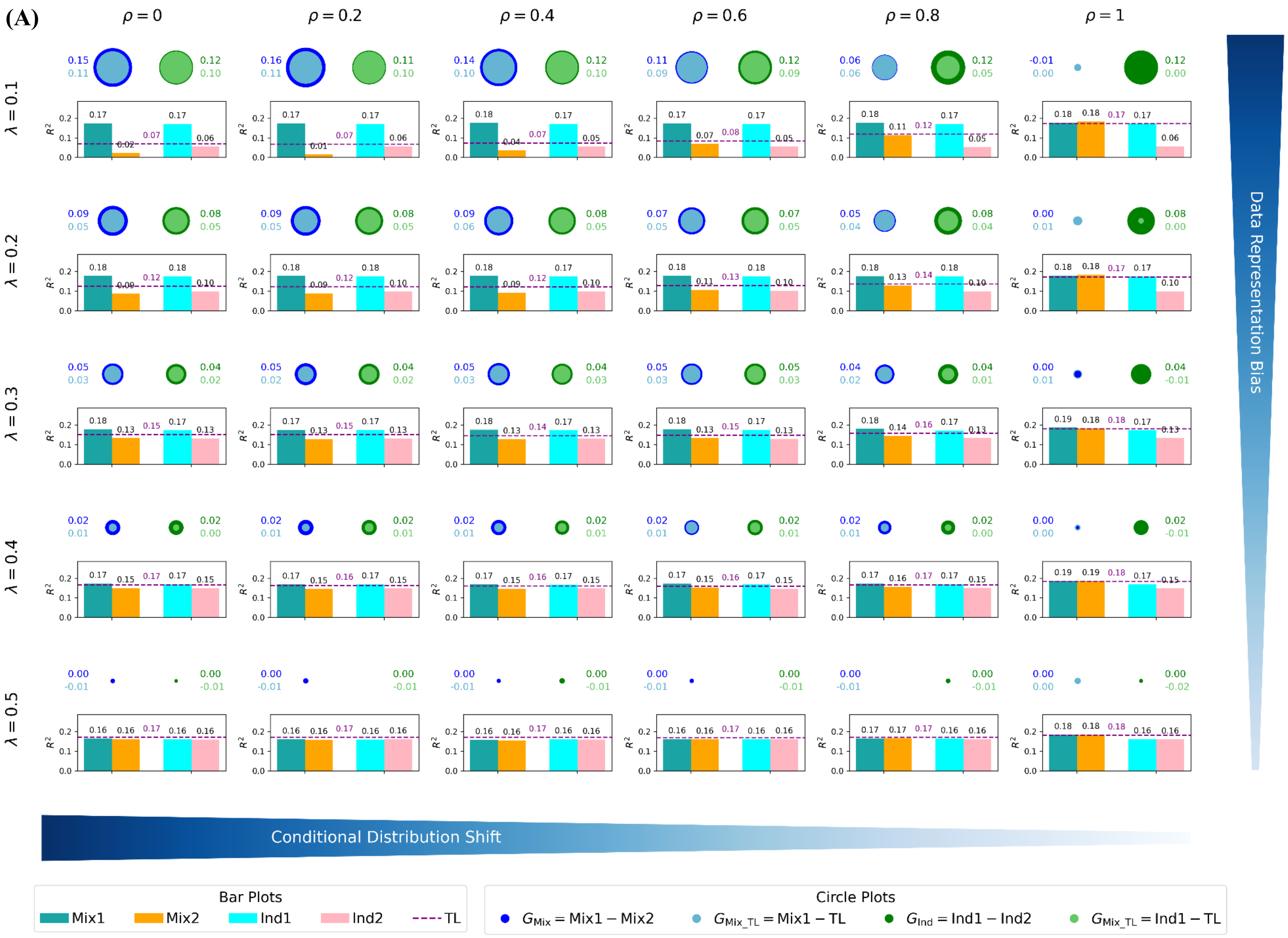
(A)
ρ = 0
ρ = 0.2
ρ = 0.4
ρ = 0.6
ρ = 0.8
ρ = 1
λ = 0.1
λ = 0.2
λ = 0.3
λ = 0.4
λ = 0.5
R²
Data Representation Bias
Conditional Distribution Shift
Bar Plots
Mix1
Mix2
Ind1
Ind2
TL
Circle Plots
G_Mix = Mix1 − Mix2
G_Mix_TL = Mix1 − TL
G_Ind = Ind1 − Ind2
G_Mix_TL = Ind1 − TL

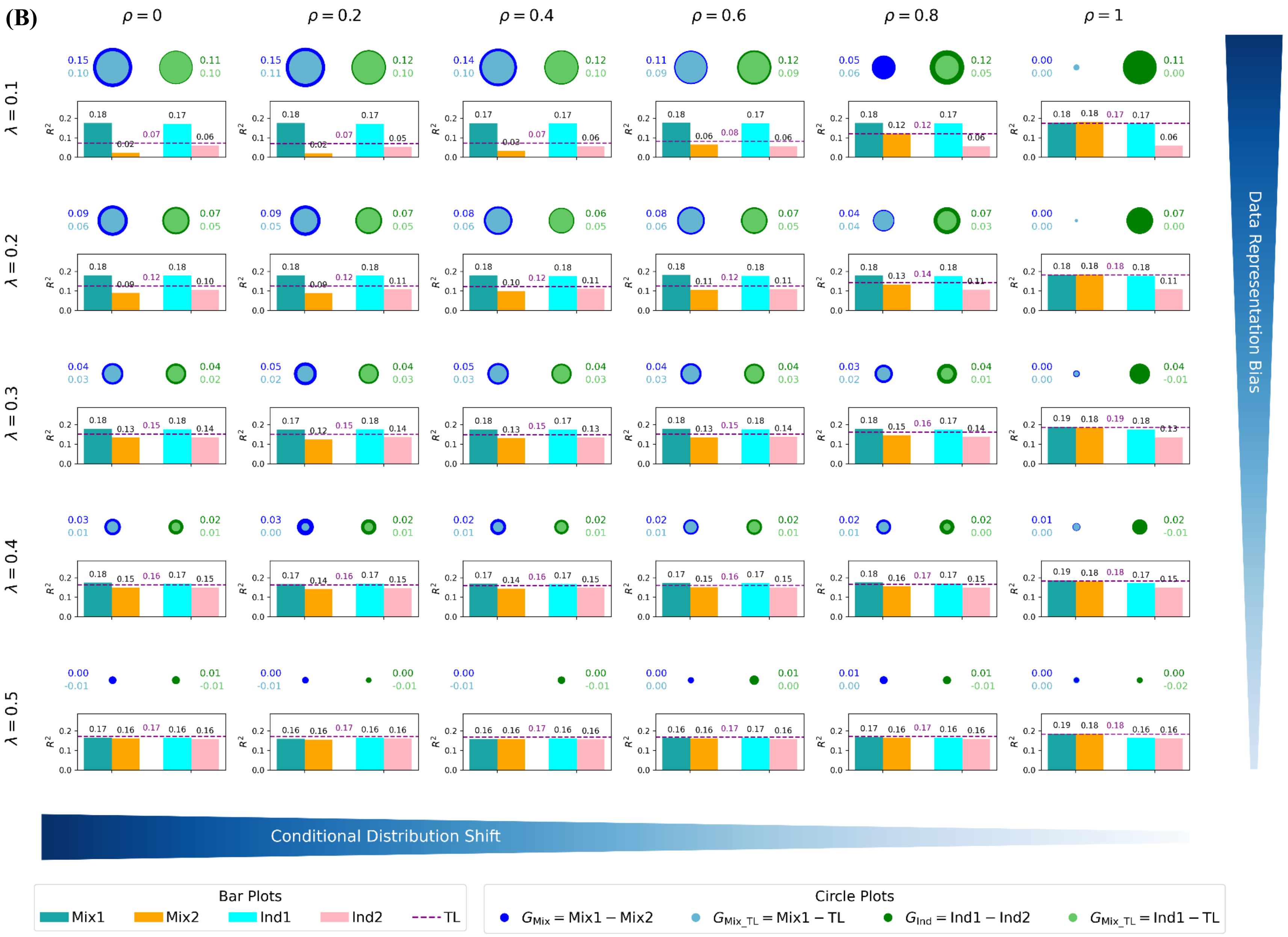
(B)
$\rho = 0$
$\rho = 0.2$
$\rho = 0.4$
$\rho = 0.6$
$\rho = 0.8$
$\rho = 1$
$\lambda = 0.1$
$\lambda = 0.2$
$\lambda = 0.3$
$\lambda = 0.4$
$\lambda = 0.5$
R^2
Data Representation Bias
Conditional Distribution Shift
Bar Plots
Mix1
Mix2
Ind1
Ind2
TL
Circle Plots
$G_{\text{Mix}} = \text{Mix1} - \text{Mix2}$
$G_{\text{Mix\_TL}} = \text{Mix1} - \text{TL}$
$G_{\text{Ind}} = \text{Ind1} - \text{Ind2}$
$G_{\text{Mix\_TL}} = \text{Ind1} - \text{TL}$

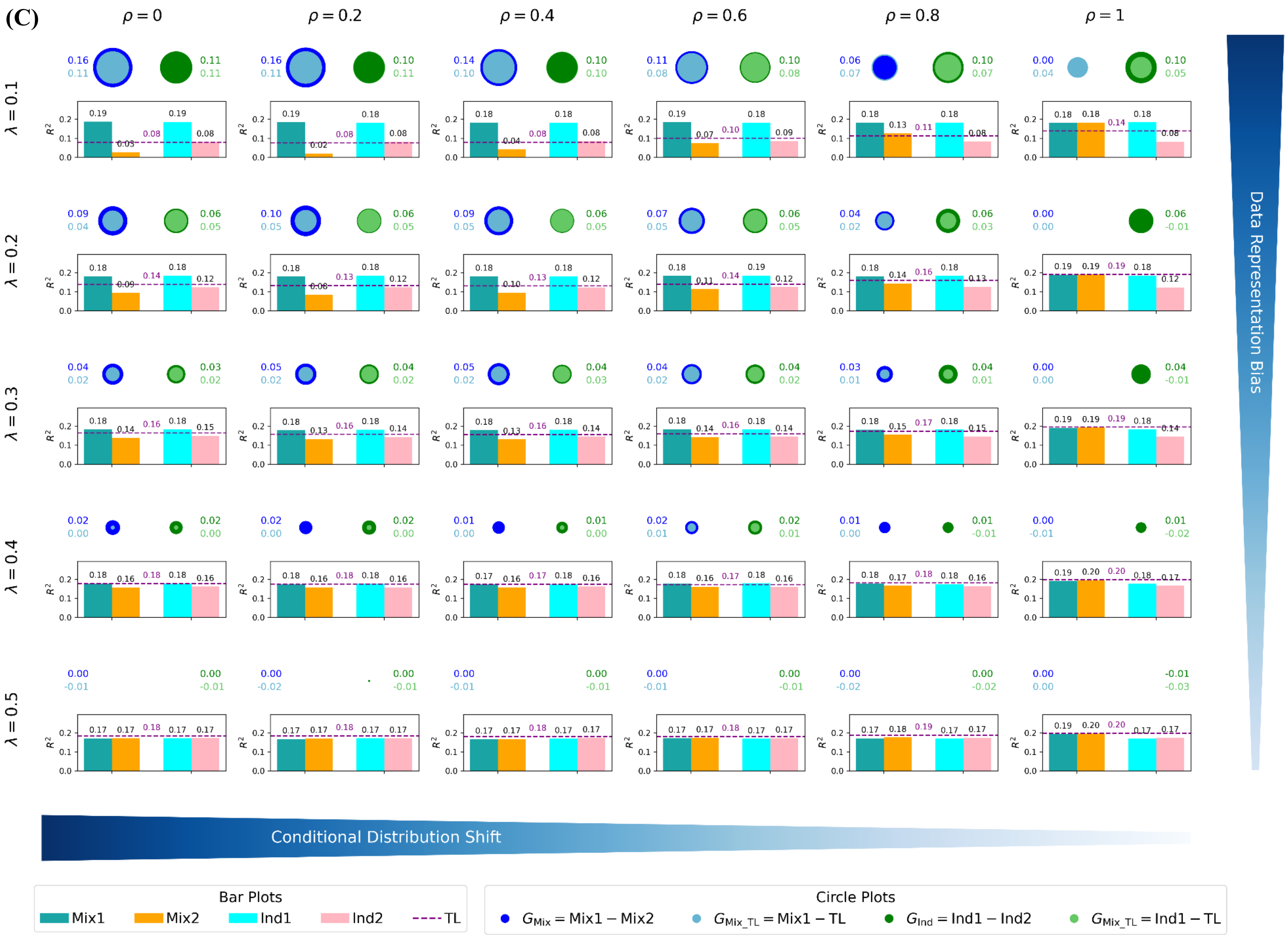
(C)
$\rho = 0$
$\rho = 0.2$
$\rho = 0.4$
$\rho = 0.6$
$\rho = 0.8$
$\rho = 1$
$\lambda = 0.1$
$\lambda = 0.2$
$\lambda = 0.3$
$\lambda = 0.4$
$\lambda = 0.5$
R^2
Data Representation Bias
Conditional Distribution Shift
Bar Plots
Mix1
Mix2
Ind1
Ind2
TL
Circle Plots
$G_{Mix} = \text{Mix1} - \text{Mix2}$
$G_{Mix\_TL} = \text{Mix1} - \text{TL}$
$G_{Ind} = \text{Ind1} - \text{Ind2}$
$G_{Mix\_TL} = \text{Ind1} - \text{TL}$

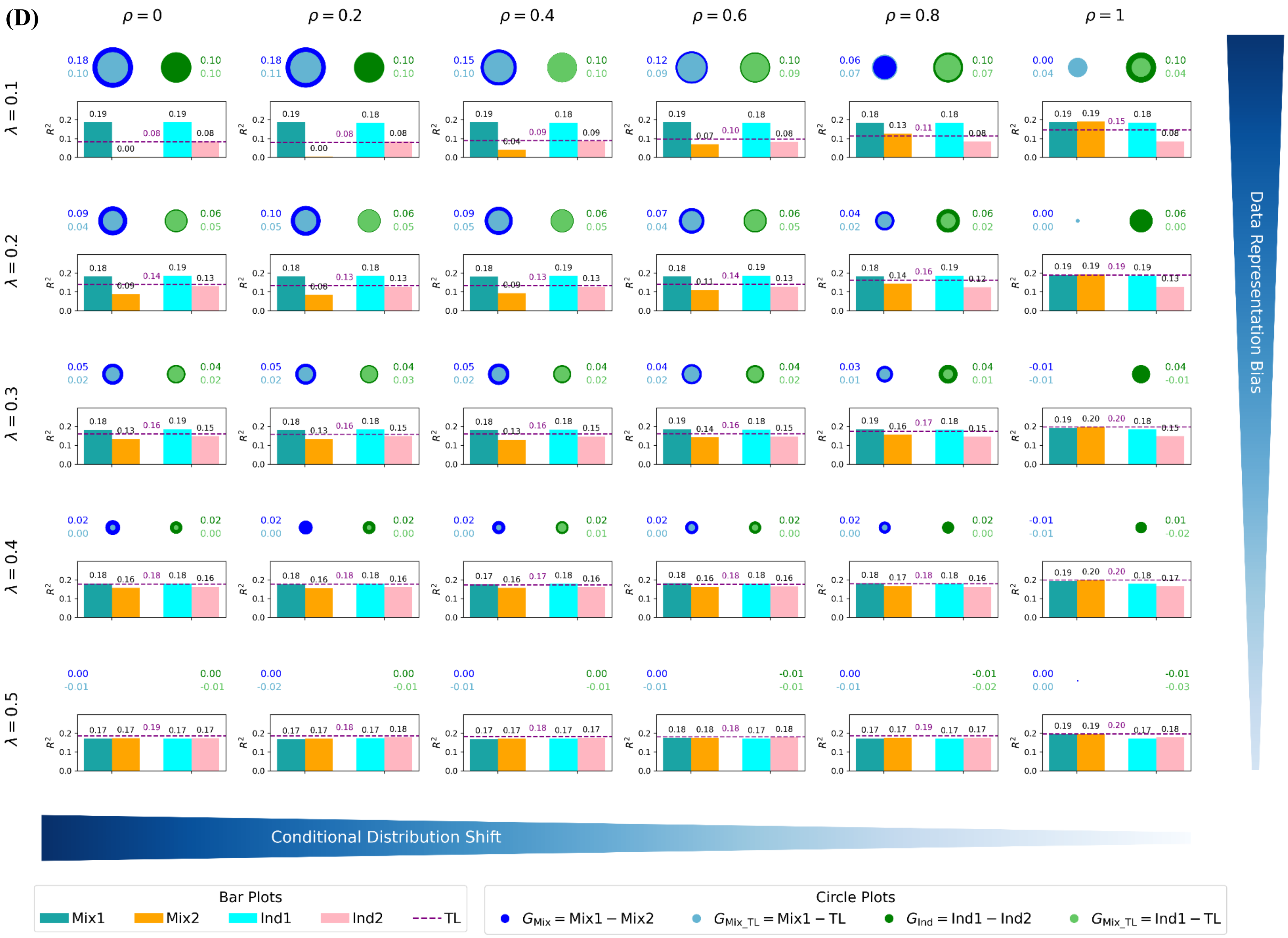
(D)
ρ = 0
ρ = 0.2
ρ = 0.4
ρ = 0.6
ρ = 0.8
ρ = 1
λ = 0.1
λ = 0.2
λ = 0.3
λ = 0.4
λ = 0.5
Data Representation Bias
Conditional Distribution Shift
Bar Plots
Mix1
Mix2
Ind1
Ind2
TL
Circle Plots
G_Mix = Mix1 − Mix2
G_Mix_TL = Mix1 − TL
G_Ind = Ind1 − Ind2
G_Mix_TL = Ind1 − TL

Fig. S1 Mapping multi-ancestry machine learning model performance landscapes for the populations consisting of **(A)** EUR and AMR, **(B)** EUR and SAS, **(C)** EUR and EAS, and **(D)** EUR and AFR (*continuous* phenotype, h^2=0.25, number of SNPs=3,000, total population size=100,000). The hierarchical grid of subplots illustrates the variation in machine learning model performance across a parameter space defined by data representation bias and conditional distribution shift. In each subplot, the bar charts and dashed line show performance of different multi-ancestry machine learning approaches. Mix1 and Mix2 represent the performance of mixture learning for EUR and DDP, Ind1 and Ind2 represent the performance of independent learning for EUR and DDP, and the dashed line represents the performance of transfer learning. The blue and light blue concentric circles represent G_{Mix} and G_{Mix_TL}, and the green and light green concentric circles represent G_{Ind} and G_{Ind_TL}.

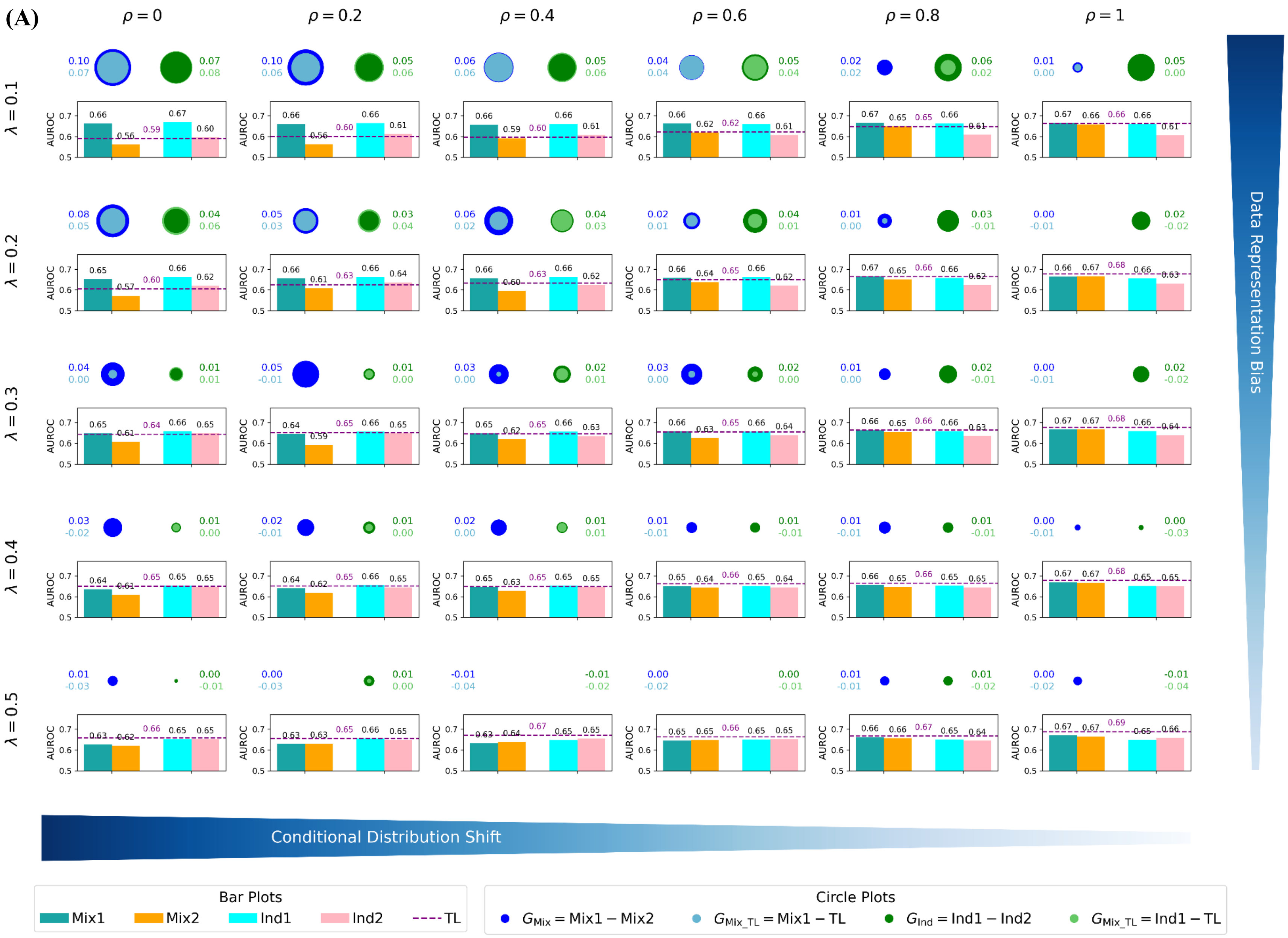

(A)
ρ = 0
ρ = 0.2
ρ = 0.4
ρ = 0.6
ρ = 0.8
ρ = 1
λ = 0.1
λ = 0.2
λ = 0.3
λ = 0.4
λ = 0.5
AUROC
Data Representation Bias
Conditional Distribution Shift
Bar Plots
Mix1
Mix2
Ind1
Ind2
TL
Circle Plots
G_{Mix} = Mix1 − Mix2
G_{Mix_TL} = Mix1 − TL
G_{Ind} = Ind1 − Ind2
G_{Mix_TL} = Ind1 − TL

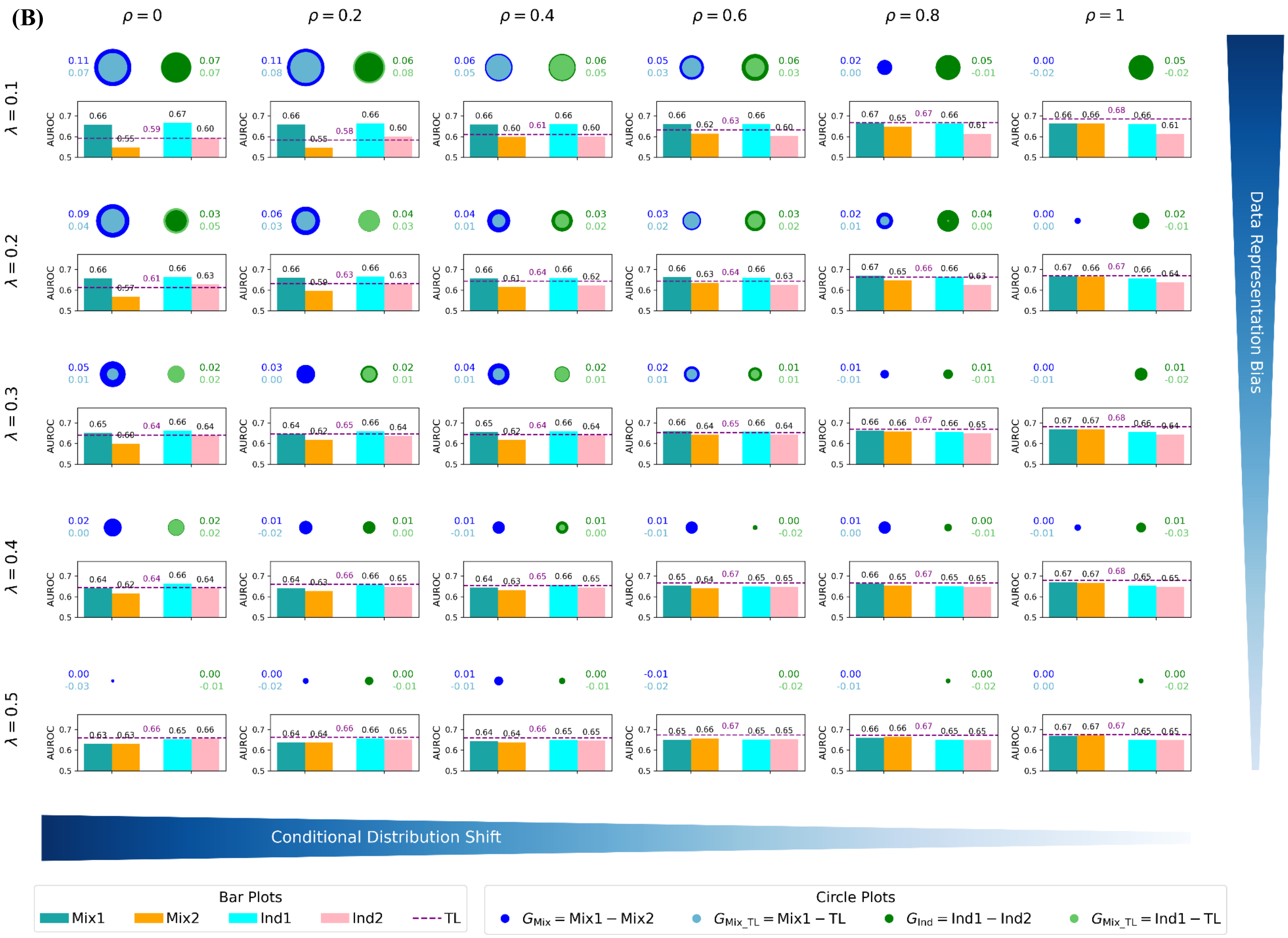
(B)
$\rho = 0$
$\rho = 0.2$
$\rho = 0.4$
$\rho = 0.6$
$\rho = 0.8$
$\rho = 1$
$\lambda = 0.1$
$\lambda = 0.2$
$\lambda = 0.3$
$\lambda = 0.4$
$\lambda = 0.5$
AUROC
Data Representation Bias
Conditional Distribution Shift
Bar Plots
Mix1
Mix2
Ind1
Ind2
TL
Circle Plots
$G_{Mix} = \text{Mix1} - \text{Mix2}$
$G_{Mix\_TL} = \text{Mix1} - \text{TL}$
$G_{Ind} = \text{Ind1} - \text{Ind2}$
$G_{Mix\_TL} = \text{Ind1} - \text{TL}$

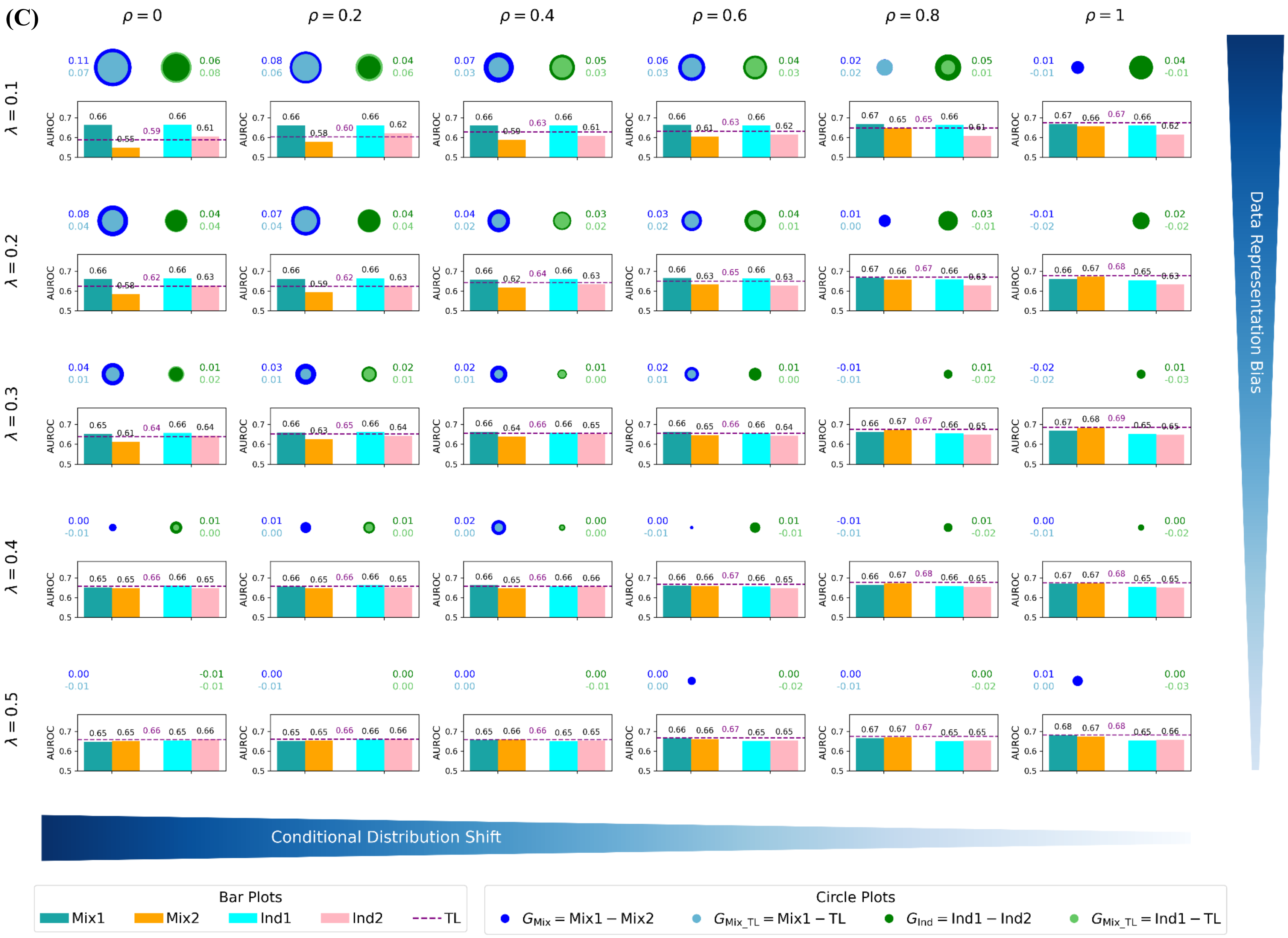
(C)
$\rho = 0$
$\rho = 0.2$
$\rho = 0.4$
$\rho = 0.6$
$\rho = 0.8$
$\rho = 1$
$\lambda = 0.1$
$\lambda = 0.2$
$\lambda = 0.3$
$\lambda = 0.4$
$\lambda = 0.5$
AUROC
Data Representation Bias
Conditional Distribution Shift
Bar Plots
Mix1 Mix2 Ind1 Ind2 TL
Circle Plots
$G_{Mix} = \text{Mix1} - \text{Mix2}$
$G_{Mix\_TL} = \text{Mix1} - \text{TL}$
$G_{Ind} = \text{Ind1} - \text{Ind2}$
$G_{Mix\_TL} = \text{Ind1} - \text{TL}$

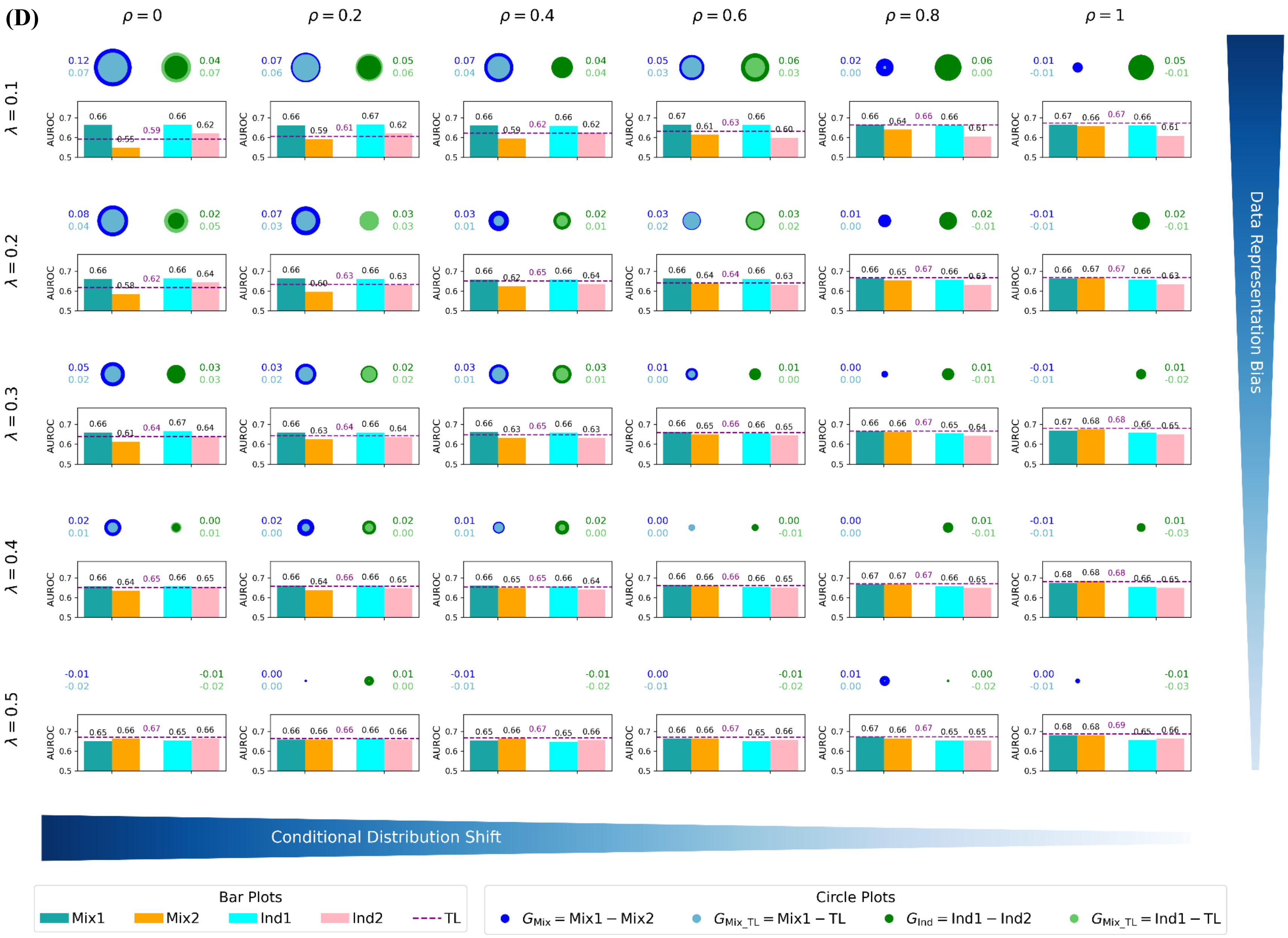
(D)
$\rho = 0$
$\rho = 0.2$
$\rho = 0.4$
$\rho = 0.6$
$\rho = 0.8$
$\rho = 1$
$\lambda = 0.1$
$\lambda = 0.2$
$\lambda = 0.3$
$\lambda = 0.4$
$\lambda = 0.5$
AUROC
Data Representation Bias
Conditional Distribution Shift
Bar Plots
Mix1
Mix2
Ind1
Ind2
TL
Circle Plots
$G_{Mix} = Mix1 - Mix2$
$G_{Mix\_TL} = Mix1 - TL$
$G_{Ind} = Ind1 - Ind2$
$G_{Mix\_TL} = Ind1 - TL$

Fig. S2 Mapping multi-ancestry machine learning model performance landscapes for the populations consisting of **(A)** EUR and AMR, **(B)** EUR and SAS, **(C)** EUR and EAS, and **(D)** EUR and AFR (*binary* phenotype, h^2=0.25, number of SNPs=500, total population size=10,000). The hierarchical grid of subplots illustrates the variation in machine learning model performance across a parameter space defined by data representation bias and conditional distribution shift. In each subplot, the bar charts and dashed line show performance of different multi-ancestry machine learning approaches. Mix1 and Mix2 represent the performance of mixture learning for EUR and DDP, Ind1 and Ind2 represent the performance of independent learning for EUR and DDP, and the dashed line represents the performance of transfer learning. The blue and light blue concentric circles represent $\mathrm{G_{Mix}}$ and $\mathrm{G_{Mix\_TL}}$, and the green and light green concentric circles represent $\mathrm{G_{Ind}}$ and $\mathrm{G_{Ind\_TL}}$.
